# Aberrant development of excitatory circuits to inhibitory neurons in the primary visual cortex after neonatal binocular enucleation

**DOI:** 10.1038/s41598-021-82679-2

**Published:** 2021-02-04

**Authors:** Rongkang Deng, Joseph P. Y. Kao, Patrick O. Kanold

**Affiliations:** 1 Department of Biomedical Engineering, Johns Hopkins University, 379 Miller Res. Bldg, Baltimore, MD 21205 USA; 2 Department of Biology, University of Maryland, College Park, MD 20742 USA; 3 Biological Sciences Graduate Program, University of Maryland, College Park, 20742 MD USA; 4 Center for Biomedical Engineering and Technology, and Department of Physiology, University of Maryland School of Medicine, Baltimore, MD 21201 USA

**Keywords:** Development of the nervous system, Neuroscience, Neural circuits

## Abstract

The development of GABAergic interneurons is important for the functional maturation of cortical circuits. After migrating into the cortex, GABAergic interneurons start to receive glutamatergic connections from cortical excitatory neurons and thus gradually become integrated into cortical circuits. These glutamatergic connections are mediated by glutamate receptors including AMPA and NMDA receptors and the ratio of AMPA to NMDA receptors decreases during development. Since previous studies have shown that retinal input can regulate the early development of connections along the visual pathway, we investigated if the maturation of glutamatergic inputs to GABAergic interneurons in the visual cortex requires retinal input. We mapped the spatial pattern of glutamatergic connections to layer 4 (L4) GABAergic interneurons in mouse visual cortex at around postnatal day (P) 16 by laser-scanning photostimulation and investigated the effect of binocular enucleations at P1/P2 on these patterns. *Gad2*-positive interneurons in enucleated animals showed an increased fraction of AMPAR-mediated input from L2/3 and a decreased fraction of input from L5/6. Parvalbumin-expressing (PV) interneurons showed similar changes in relative connectivity. NMDAR-only input was largely unchanged by enucleation. Our results show that retinal input sculpts the integration of interneurons into V1 circuits and suggest that the development of AMPAR- and NMDAR-only connections might be regulated differently.

## Introduction

Cortical circuits require intricate connections among different types of neurons. For the initial assembly of cortical circuits, genetically programed molecular cues are essential for neurogenesis, cell migration, synapse formation and axonal guidance^1–3^. The sensory epithelium also influences the development of cortical circuits, likely through neural activity-dependent mechanisms^4^.

The development of visual system is heavily influenced by input from the retina^5^. Complete removal of retinae early during development leads to abnormal expression of cortical areal patterning genes^6^, suggesting a reorganization of functional areas in the cortex. Moreover, neurons in the visual cortex develop increased responses to other sensory modalities^7–9^, and visual cortical neurons receive increased intracortical and thalamocortical connections from brain areas that normally do not innervate visual cortex^10–12^. Retinotopically-matched callosal connections in the visual cortex fail to develop after neonatal enucleation^13–15^. Thus, losing retinal input causes large-scale reorganization of long-distance connections to neurons in the visual cortex. The visual cortex also has extensive translaminar local connections to both excitatory and inhibitory neurons^16,17^, but it remains unknown whether the early development of translaminar local circuits in visual cortex requires retinal input.

The translaminar circuits impinging on cortical γ-aminobutyric acid (GABA)-ergic interneurons undergo extensive changes during the early postnatal development in mice. GABAergic interneurons arrive at their final destinations in the cortex later than cortical excitatory neurons^18–20^. Connections from cortical excitatory neurons to GABAergic interneurons are mediated by glutamate receptors including ionotropic α-amino-3-hydroxy-5-methyl-4-isoxazolepropionic acid (AMPA) and *N*-methyl-D-aspartate (NMDA) glutamate receptors. Although GABAergic interneurons have functioning neurotransmitter receptors early during development, they receive glutamatergic input only from a few nearby excitatory neurons by the end of migration (postnatal day (P) 6 in mice)^21–23^, predominantly mediated by NMDARs^23^. Subsequently, GABAergic interneurons gradually receive more glutamatergic inputs from distant excitatory neurons^21–23^, and the ratio of NMDAR-only to AMPAR-mediated inputs decreases^23,24^. Important developmental processes such as critical period plasticity in the visual cortex rely on the development of cortical inhibition^25–28^, but little is known about the factors regulating the early development of circuits to GABAergic interneurons in visual cortex.

Accordingly, we investigated a possible role for retinal input in the development of the translaminar circuits connecting GABAergic interneurons in the primary visual cortex (V1). We removed retinae in neonatal mice and compared the spatial pattern of glutamatergic connections received by layer 4 (L4) *Gad2* and PV interneurons at P16/17 after the completion of eye opening and maturation of V1 firing patterns^29^. We used glutamate uncaging via laser-scanning photostimulation (LSPS) combined with whole-cell patch clamp recording, finding that inhibitory neurons from enucleated animals showed a decreased proportion of AMPAR-mediated connections from subgranular layers and an altered spatial distribution of AMPAR-mediated connections. Glutamate uncaging revealed NMDAR-only connections to GABAergic interneurons were largely unaffected by enucleation. We conclude that peripheral input is required for the normal development of the translaminar circuits to L4 GABAergic interneurons in V1 during early ages.

## Materials and methods

All procedures were approved by the University of Maryland Institutional Animal Care and Use Committee to be in accordance with applicable guidelines and regulations. The study was carried out in compliance with the ARRIVE guidelines.

### Animals and binocular enucleation

*Gad2-Cre* mice (010802, Jackson Laboratories)^30^, *Pvalb-*Cre mice (008069, Jackson Laboratories)^31^ crossed with *Ai9* Cre-reporter transgenic mice (007909, Jackson Laboratories)^32^ were used to generate pups with red fluorescent protein expression in GABAergic interneurons (Fig. 2A and 6A) for all experiments. Mouse pups of both sexes were enucleated at postnatal day 1 (P1) or P2. Pups were anesthetized by hypothermia, eyelids were opened with fine scissors, and eyes were removed with fine forceps. The eye lids were closed with surgical glue. Control pups only experienced similar hypothermia at the same age. Pups recovered on a heating pad before returning to their home cage.

### Electrophysiology

Coronal slices (400 µm) from control and enucleated animals at P16/P17 were used for all recordings. Neurons in the binocular region of the V1 were selected for recordings. For each experimental group, at least three animals were used for making brain slices. Typically, we recorded from two brain slices from each animal. Slices were cut with a vibratome (VT1200, Leica) in ice cold modified artificial cerebrospinal fluid (ACSF) containing (in mM): 212.7 sucrose, 2.6 KCl, 1.23 NaH_2_PO_4_, 26 NaHCO_3_, 10 glucose, 3 MgCl_2_, 1 CaCl_2_ (pH 7.35–7.4). Slices were incubated at 30 °C for 30 min, then kept at room temperature in ACSF containing (in mM): 130 NaCl, 3 KCl, 1.25 NaHCO_3_, 10 glucose, 1.3 MgSO_4_ and 2.5 CaCl_2_ (pH 7.35–7.4). All ACSF were equilibrated with 95% O_2_-5% CO_2_. Fluorescence-targeted recordings were performed as previously^23^; borosilicate recording electrodes (4–9 MΩ) were filled with internal solution containing (in mM): 115 cesium methanesulfonate, 5 NaF, 10 EGTA, 10 HEPES, 9 CsCl, 3.5 MgATP, 0.3 NaGTP and 3 QX-314 (pH 7.25; 300 mOsm); 0.5% Biocytin was added to the internal solution. Red fluorescent protein-expressing interneurons in L4 of the binocular region of V1 were identified with epifluorescence microscopy. Recordings were performed at room temperature in high-divalent ACSF containing (in mM): 124 NaCl, 5 KCl, 1.23 NaH_2_PO_4_, 26 NaHCO_3_, 10 glucose, 4 MgCl_2_ and 4 CaCl_2_. Picrotoxin (100 μM) was added to the bath solution during recordings to block GABA_A_ receptor-mediated currents^23^. All drugs and chemicals were purchased from Sigma-Aldrich unless specified otherwise. Data were acquired with a voltage-clamp amplifier (Multiclamp 700B; Molecular Devices) and a DAQ board (NI PCI-6259, National Instruments) controlled by EPHUS software^33^ running in MATLAB (The Mathworks). Membrane potential was corrected with 10 mV of estimated liquid junction potential. Series resistance was typically between 20 and 40 MΩ. Input resistance was similar between control and enucleation conditions.

### Laser-scanning photostimulation (LSPS)

LSPS was performed as described previously^23,34^. High-divalent ACSF containing 0.8 mM caged glutamate [*N*-(6-nitro-7-coumarylmethyl)-l-glutamate]^35,36^ was used as the bath solution during recording. UV laser (3510-100, DPSS Lasers Inc.) stimulation (355 nm, 1-ms pulses) was delivered through a 10 × water immersion objective (Olympus UMPLFLN10XW). Laser-scanning directions were controlled by a mirror galvanometer (6210H, Cambridge Technology). Laser power on the specimen was between 22 and 25 mW. For each map, a rectangular array of up to 30 × 30 sites with 40 μm spacing was stimulated once at 1 Hz in a pseudorandom order. This stimulation paradigm evokes action potentials in neurons at the stimulation sites with similar spatial resolution (~ 100 μm) in all cortical layers between the control and enucleation groups. Putative monosynaptic currents (EPSCs) in GABAergic interneurons were classified by the latency of the evoked currents. We interpreted EPSCs with latencies less than 10 ms as the results of direct activation of the glutamate receptors on the patched cell. EPSCs with latencies between 10 and 50 ms are interpreted as monosynaptic EPSCs (Fig. 2A). A minimal peak amplitude of 10 pA was used to minimize false positive EPSCs. The peak amplitude and the transferred charge (the area under the EPSC trace between 10 and 50 ms) of the EPSCs were used as measures of connection strength. Based on previous experiments using TTX, we estimate that 90% of the classified monosynaptic EPSC events were correctly identified using these event criteria^23,34^. For each neuron, recordings were made at − 70 mV and + 40 mV to map, respectively, AMPAR-mediated inputs and to include NMDAR-mediated current for each stimulation site. Input maps obtained at + 40 mV and − 70 mV holding potential were compared for each cell: at any spatial location, an input that was present only at + 40 mV was identified as NMDAR-only input (Fig. 4A,B). A given presynaptic neuron may make multiple synapses with a postsynaptic cell and only some of them might be NMDAR-only synapses. Our method cannot resolve such a scenario and would treat this connection as AMPAR-mediated. Thus, on the individual synaptic level we may underestimate the number of NMDAR-only synapses.

**Figure 1:**
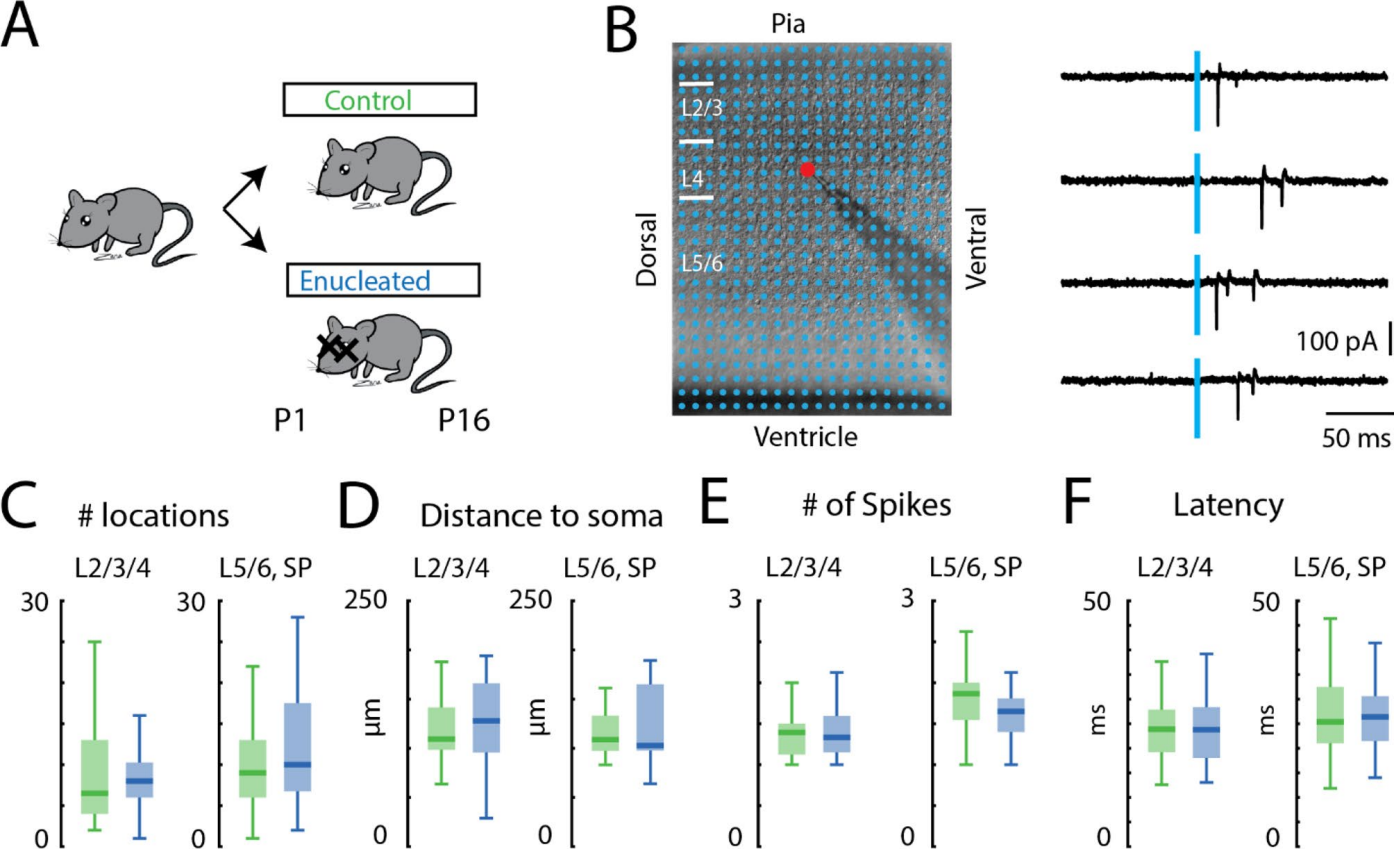
Spiking responses to glutamate photostimulation are not changed in enucleated animals. (**A**) Binocular enucleation is performed at P1/P2 and the glutamatergic connections received by GABAergic interneurons are studied at P16/P17. (**B**) An example LSPS experiment in a coronal slice of V1. LSPS stimulation locations are marked as blue dots on the DIC image. The red dot indicates soma location of the recorded *Gad2* interneuron. White bars mark layer boundaries. The length of the white bars is 100 μm. Example traces of action potentials in response to glutamate photostimulation through cell-attached recording. Blue line indicates uncaging time point. (**C**) Boxplot of the number of locations where action potentials can be evoked by glutamate photostimulation of the cortical excitatory neurons. P > 0.05. (**D**) Boxplot of the distance within which 80% of action potentials were evoked in cortical excitatory neurons. P > 0.05. (**E**) Boxplot of the average number of action potentials evoked by glutamate photostimulation of the cortical excitatory neurons. P > 0.05. (**F**) Boxplot of the average first action potential latency evoked by glutamate photostimulation of the cortical excitatory neurons. P > 0.05. For (**C–F**) L2/3/4: green, control n = 22; blue, enucleation n = 21. L5/6 and SP: green, control n = 22; blue, enucleation n = 25. Statistics for (**C**–**F**) are in Table 1.

It is possible that binocular enucleation could have introduced broad excitability changes in neurons that would have influenced the efficacy with which uncaged glutamate would evoke action potentials, subsequently changing the spatial resolution of our stimulation method. To examine this, we used loose-patch recordings to record action potentials evoked by photostimulation^37^ (Fig. 1). Patch pipettes were filled with filtered high-divalent ACSF. Typically, 1–2 action potentials were reliably evoked by our stimulation from sites within 150 μm of the cell body (Fig. 1D,E).

### Data analysis and statistics

**Figure 2:**
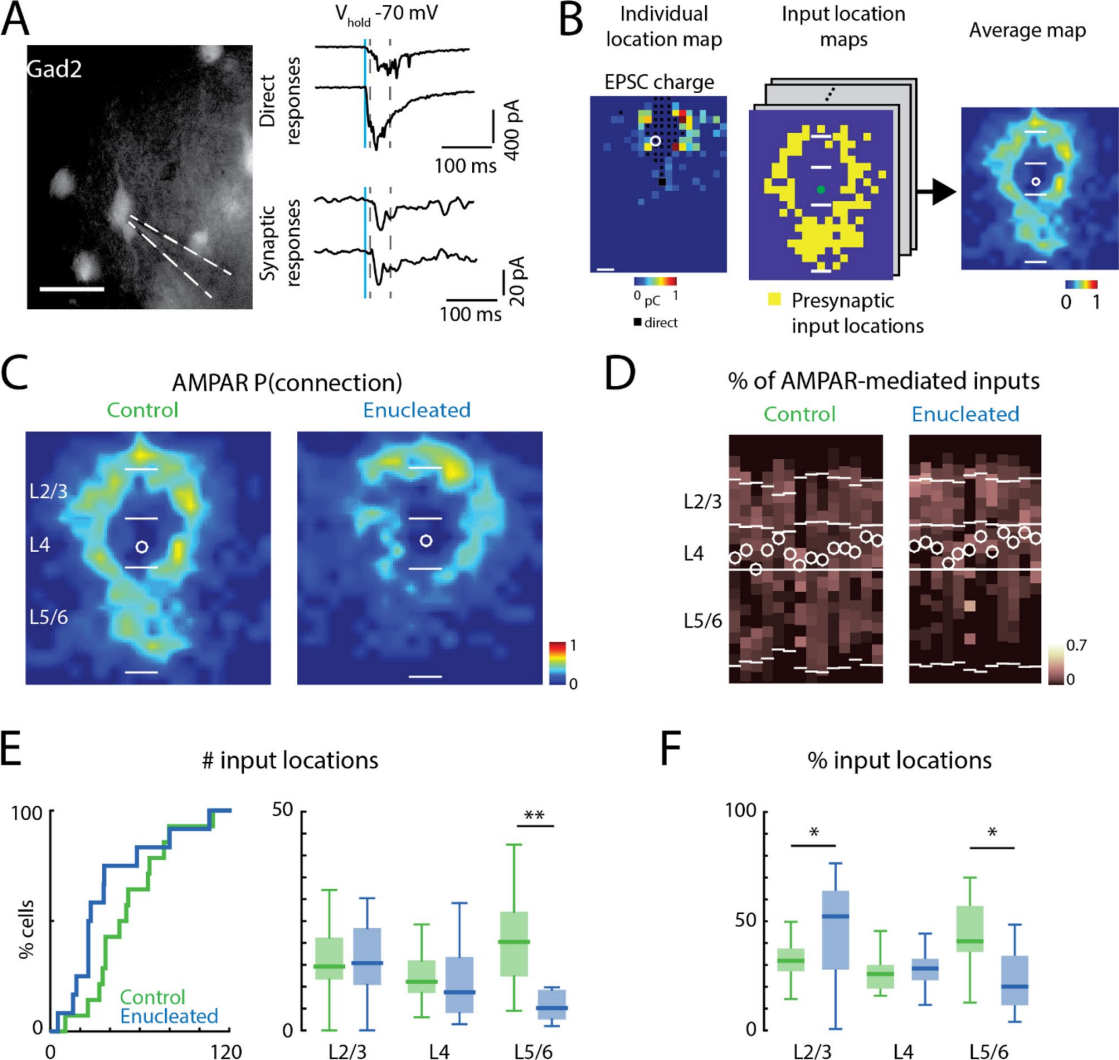
AMPAR-mediated cortical inputs to *Gad2* interneurons from subgranular layers are reduced after enucleation. (**A**) An example *Gad2-Cre::tdTomato* interneuron under epifluorescence microscopy. Scale bar = 50 µm. Traces show example recordings of responses to direct photostimulation (upper traces) and responses to activation of presynaptic cells (lower traces). Blue line indicates photostimulation time point. Dashed lines indicate the time window (10 to 50 ms post-photostimulation) for identifying putative monosynaptic responses. (**B**) An exemplar input location map from LSPS experiments (left). The connected locations are color-coded based on the charge of the recorded EPSCs. Locations with black squares are affected by direct activation of the recorded neuron and are excluded in further analysis. White circle indicates the location of the recorded neuron. The locations of presynaptic cells are reconstructed into input maps (middle). Input maps are aligned to the soma and averaged to generate average input maps (right). (**C**) Average spatial connection probability maps of AMPAR-mediated inputs to L4 *Gad2* interneurons in V1 at P16/P17 in control and enucleated animals (enucleated at P1/P2) (n = 14 control and n = 12 enucleated). Maps show the fraction of cells that received an input from a particular spatial location. AMPAR-mediated inputs from subgranular layers are decreased after enucleation. The length of the scale bars marking the layer boundaries is 100 μm. (**D**) Laminar distribution of inputs for each cell. Plotted is the fraction of input each cell received at each laminar location. Layer borders for each cell are indicated by horizontal white lines. Soma locations are indicated by white circles; cells are aligned to L4. (**E**) Quantification of the number of input locations. Cumulative distribution function (CDF) of the total number of input locations (left). P > 0.05. Boxplot (right) shows the number of input locations in each layer group. AMPAR-mediated inputs from L5/6 are decreased after enucleation. (**) indicates P < 0.01, otherwise P > 0.05. (**F**) Boxplot shows the average fraction of inputs from each layer. The fraction of L5/6 inputs is decreased after enucleation. (*) indicates P < 0.01, otherwise P > 0.05. For all panels: green, control group; blue, enucleation group. Statistics for (**E**) (left) are in the main text. Statistics for (**E**) (right) and (**F**) are in Table 2.

Data were analyzed in MATLAB with custom routines. Features in the bright-field image were used to identify cortical layer boundaries as previously described^34,38,39^. Monosynaptically-evoked EPSCs were distinguished from directly-evoked EPSCs by a latency threshold as detailed above. We then quantified the number of stimulation locations that give rise to synaptic EPSCs and divided them based on their layer of origin. The fractional input from each layer group was the number of stimulation locations that could evoke synaptic EPSCs from each layer group divided by the total number of stimulation locations that could evoke synaptic EPSCs from all layers. To reveal the general pattern of the input locations, we aligned input maps with the soma of the recorded neuron and calculated the fraction of cells that received an input from each stimulation location (Fig. 2B) and the average EPSC strength. The locations close to the soma and dendrites of the recorded cells were not included in the analysis due to direct activation (Fig. 4B). Therefore, the average input maps had a black central zone. All average connection maps had the same orientation, with pia side on the top and ventricle side at the bottom.

**Figure 3:**
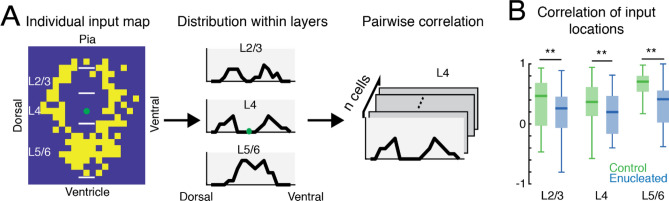
Decreased variability of the input locations between cells after enucleation. (**A**) Quantification of the variability of the input locations. The distribution profiles of input from each layer group are calculated from input location maps. Pairwise correlation is calculated from the distribution profiles from different cells to quantify the variability of the input locations. (**B**) Boxplot of the correlation of input locations originating from each layer. (**) indicates P < 0.01, otherwise P > 0.05. Statistics for (**B**) are in Table 3.

Changes in the input locations can also lead to changes in the average input location map even without significant changes in the input area. Inputs consistently from the same location for all cells will increase the probability of finding presynaptic input in that location on the average input location map. Inconsistent input locations will lower the probability of finding presynaptic inputs in the average input location map. This reflects how consistent the spatial distribution of the inputs is from cell to cell. To quantify the consistency of the spatial distribution of the inputs, for each cell, we calculated the distribution of the input locations in 80 μm bins along the medial to lateral axis in each layer group. For each layer group, we use the distributions from any combination of 2 cells in the same experimental group to calculated Pearson's Linear Correlation Coefficient as a measure of input location variability (Fig. 3A). Consistent input locations will generate similar distribution and the pairwise correlation coefficients were closer to 1. Pairwise correlation coefficients were subsequently compared between the control and enucleated conditions.

For group comparisons we first used a Shapiro-Wilks test to evaluate if data were normally distributed. If both groups were normally distributed, a two-sample *t*-test was used to test the significance of the difference. Otherwise, a Wilcoxon rank-sum test was used. Significant differences were marked as (*) P < 0.05 or (**) P < 0.01. Comparisons with P > 0.05 were not marked in the figures. Median and interquartile range (IQR) were reported for each data group. Cohen’s d and r were used to quantify the effect size for two-sample *t*-test and Wilcoxon rank-sum test, respectively. Cohen’s d values larger than 0.8 indicates large effect. r values larger than 0.5 indicates large effect. Descriptive statistics and statistical test methods were detailed in the main text and the tables.

## Results

We sought to determine if retinal input is required for the normal maturation of glutamatergic connections received by layer 4 (L4) GABAergic interneurons in V1. We thus performed bilateral enucleation in mouse pups at P1/P2 when GABAergic interneurons receive few glutamatergic connections from cortical excitatory neurons^21^. As cortical firing patterns in V1 mature at ~ P16/17^29^ just before the onset of the critical period^40–42^, we assessed the effect of neonatal enucleation at P16/P17. The cortical sources of glutamatergic connections to L4 GABAergic interneurons were mapped by laser-scanning photostimulation (LSPS) using caged glutamate and whole-cell patch clamp recordings in acute brain slices in vitro^23,34^.

We first tested if enucleation changed cortical neurons’ responses to glutamate uncaging, thereby changing the spatial resolution and efficacy of our LSPS technique. Thus, we performed cell-attached recordings from excitatory neurons in V1 from all cortical layers (control: L2/3/4, n = 22 cells, L5/6 and SP, n = 22 cells; enucleation: L2/3/4, n = 22 cells, L5/6 and SP, n = 25 cells) during glutamate photostimulation (Fig. 1B). We found that enucleation did not alter the total number of stimulus locations from which action potentials could be evoked (Fig. 1C; Table 1), the distance of stimulation locations where action potentials can be evoked (Fig. 1D; Table 1), the average number of evoked action potentials (Fig. 1E; Table 1), nor the average latency of the evoked action potentials (Fig. 1F; Table 1). Thus, the excitation pattern of the excitatory neurons in response to photostimulation and the spatial resolution of LSPS in V1 were comparable between control and enucleated animals in our experimental paradigm.

**Table 1:** Statistics in Fig. 1.

Figure 1C: total number of effective stimulus locations							
Layer groups	Control		Enucleation		P	Effect size	Test method
	Median	IQR	Median	IQR			
L2/3/4	6.5	9	8	4.25	0.4	0.13	Wilcoxon rank-sum test
L5/6, SP	9	7	10	10.75	0.306	0.3	Two-sample *t*-test

Figure 1D: distance to soma							
Layer groups	Control		Enucleation		P	Effect size	Test method
	Median (µm)	IQR	Median (µm)	IQR			
L2/3/4	109	42	128	70	0.388	0.13	Wilcoxon rank-sum test
L5/6, SP	108	35	102	67	0.725	0.05	Wilcoxon rank-sum test

Figure 1E: average number of spikes							
Layer groups	Control		Enucleation		P	Effect size	Test method
	Median	IQR	Median	IQR			
L2/3/4	1.4	0.38	1.3	0.4	0.884	0.02	Wilcoxon rank-sum test
L5/6, SP	1.8	0.5	1.6	0.4	0.051	0.59	Two-sample *t*-test

Figure 1F: average latency of the first spike							
Layer groups	Control		Enucleation		P	Effect size	Test method
	Median (ms)	IQR	Median (ms)	IQR			
L2/3/4	24	8.6	23	10	0.8	0.04	Wilcoxon rank-sum test
L5/6, SP	25	11	26	9	0.94	0.01	Wilcoxon rank-sum test

*IQR* interquartile range.

### Proportion of AMPAR-mediated inputs from subgranular layers decreased in *Gad2* interneurons after enucleation

We compared the source of glutamatergic inputs to L4 GABAergic interneurons in V1 in slices at P16/17 from control and enucleated animals. To perform targeted recordings from GABAergic interneurons, we used the offspring of *Gad2-Cre* (a pan-interneuron marker)^30,43^ and floxed-tdTomato transgenic mice (Ai9) to drive red fluorescent protein expression in *Gad2*-positive GABAergic interneurons (Fig. 2A). The source of glutamatergic connections received by the fluorescent GABAergic interneurons was identified by LSPS glutamate uncaging and whole-cell patch clamp recording (control: n = 14 cells, enucleation: n = 12 cells).

For each recorded GABAergic interneuron, we measured AMPAR-mediated EPSCs at a holding potential of – 70 mV while using LSPS glutamate uncaging to stimulate cortical neurons (Fig. 2A). The stimulation grid covered a cortical area of 0.8 × 1 mm^2^ around the recorded neuron, encompassing all cortical layers (Fig. 1B). For each recorded neuron, we generated an input map showing the locations of the presynaptic inputs by using the existence of monosynaptic EPSCs as an indicator of presynaptic inputs from the stimulation locations (Fig. 2B). Then we aligned the somata of all recorded neurons under each condition and averaged the input location maps to generate a 2-dimensional map reflecting the spatial probability of connections in each recorded population (Fig. 2B,C). By averaging the input location maps along the dorsal to ventral dimension we generated a laminar input probability plot (Fig. 2D). Qualitative comparison of both plots between control and enucleated animals suggested that cells in enucleated animals had fewer AMPAR-mediated inputs from subgranular layers L5/6 (Fig. 2C,D). To confirm these qualitative observations, we next performed analysis on a single cell basis. We quantified the total number of locations of AMPAR-mediated inputs to each cell by summing the stimulation sites that resulted in an evoked EPSC (Fig. 2E, left). We found that there was a trend toward a decreased total number of AMPAR-mediated input locations (control median: 48, IQR: 31, enucleation median: 26, IQR: 25, P = 0.117, effect size: r = 0.31, Wilcoxon rank-sum test). Given that the average and laminar maps (Fig. 2C,D) suggested that differences were largest for L5/6, we next performed a laminar analysis. Separately comparing the number of input locations from each layer revealed that there were fewer AMPAR-mediated input locations from L5/6 in cells from enucleated animals than in control (Fig. 2E, right; Table 2). Accordingly, the fraction of AMPAR-mediated input locations from L5/6 was lower in cells from enucleated animals than in control, while the fraction of inputs from L2/3 was increased in cells from enucleated animals (Fig. 2F; Table 2). In contrast, the total number of AMPAR-mediated input locations originating from L2/3 and L4 was not altered by enucleation.

**Table 2:** Statistics in Fig. 2.

Figure 2E: average number of effective stimulus locations in each layer group							
Layer groups	Control		Enucleation		P	Effect size	Test method
	Median	IQR	Median	IQR			
L2/3	14.6	9.5	15.4	13	0.982	0.01	Two-sample *t*-test
L4	11.1	7.4	8.7	12.8	0.793	0.11	Two-sample *t*-test
L5/6	20.2	14.7	5.1	6.7	0.008	0.52	Wilcoxon rank-sum test

Figure 2F: percentage of input in each layer group							
Layer groups	Control		Enucleation		P	Effect size	Test method
	Median	IQR	Median	IQR			
L2/3	0.319	0.104	0.522	0.36	0.049	0.88	Two-sample *t*-test
L4	0.258	0.109	0.283	0.097	0.355	0.38	Two-sample *t*-test
L5/6	0.409	0.211	0.201	0.226	0.011	1.12	Two-sample *t*-test

*IQR* interquartile range.

Besides causing a reduced number of input locations from L5/6, enucleation could subtly alter the spatial pattern of AMPAR-mediated input locations. In particular, while the probability of presynaptic cell location in the average maps suggested differences in L2–4, we did not find a difference in the number of AMPAR-mediated input locations from L2–4. Since heterogeneity in the spatial pattern between cells could lead to reduced probability of presynaptic cell location in the averaged map, we next investigated if the locations of the presynaptic connections were more variable between the control and enucleation condition. To quantify such spatial changes, we calculated the marginal distribution of the AMPAR-mediated connections along the dorsal—ventral axis (thus parallel to the pia) for cells in each layer group and then tested how similar this marginal distribution was within each group (Fig. 3A). To quantify similarity, we computed the pairwise correlation between cells within the control and enucleation group (Fig. 3A). Higher pairwise correlations indicate that the marginal distributions of the connections along the dorsal–ventral (intralaminar) axis are more similar between the cells. Such similarity would give rise to a higher connection probability and more color-intense area on the average input location probability maps. This analysis showed that cells from enucleated animals showed lower correlations of the input locations in all cortical layers than cells from control animals (Fig. 3B; Table 3). These results suggest an increase in heterogeneity in the intracortical AMPAR-mediated circuits impinging on L4 GABAergic interneurons after enucleation. Thus, our data suggest that normal maturation and spatial refinement of AMPAR-mediated inputs to GABAergic interneurons in L4 of V1 requires peripheral input.

**Table 3:** Statistics in Fig. 3.

Figure 3B: correlation of effective stimulus locations in each layer group							
Layer groups	Control		Enucleation		P	Effect size	Test method
	Median	IQR	Median	IQR			
L2/3	0.466	0.713	0.257	0.52	0.0085	0.22	Wilcoxon rank-sum test
L4	0.365	0.481	0.197	0.632	0.0035	0.23	Wilcoxon rank-sum test
L5/6	0.7	0.263	0.411	0.532	3.39 × 10^–11^	0.53	Wilcoxon rank-sum test

IQR: interquartile range.

### NMDAR-only connections are not altered in *Gad2* interneurons after enucleation

**Figure 4:**
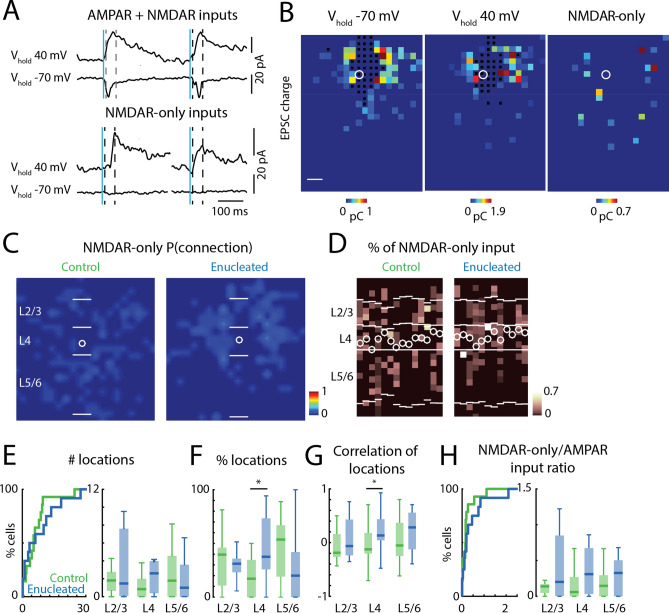
NMDAR-only cortical inputs to *Gad2* interneurons. (**A**) Example recordings of AMPAR-mediated inputs (upper traces) and NMDAR-only inputs (lower traces). Blue line indicates photostimulation time point. Dashed lines indicate the time window (10 to 50 ms post-uncaging) for identifying putative monosynaptic responses. (**B**) Example input maps from one recorded GABAergic interneuron. Responses to photostimulation are reconstructed based on the stimulation locations. Left, AMPAR-mediated input map; middle, AMPAR + NMDAR input map; right, NMDAR-only input map. Monosynaptic responses are color-coded based on the EPSC transferred charge (pseudocolor scale below each panel). Black squares indicate direct responses of the patched neuron to photosimulation. The white circle indicates soma location. Slice orientation is the same as in Fig. 1B. Scale bar is 100 μm. (**C**) Average spatial connection probability maps of NMDAR-only inputs received by L4 *Gad2* interneurons in V1 at P16/P17 in control and enucleated animals (n = 14 control and n = 12 enucleated, same cells as in Fig. 2C). (**D**) Laminar distribution of NMDAR-only inputs for each cell. Plotted is the fraction of input each cell received at each laminar location. Layer borders for each cell are indicated by horizontal white lines. Soma locations are indicated by white circle; cells are aligned to L4. (**E**) Quantification of the number of input locations. CDF of the total number of input locations (left). P > 0.05. Boxplot shows the number of input locations in each layer group (left). All P > 0.05. (**F**) Boxplot of the average fraction of inputs from each layer. (*) indicates P < 0.05, otherwise P > 0.05. (**G**) Boxplot of the correlation of input locations from each layer originate. (*) indicates P < 0.05, otherwise P > 0.05. (**H**) Quantification of NMDAR-only inputs to AMPAR-mediated inputs ratio for each cell (left) and for each layer group (right). P > 0.05. For all panels: green, control group; blue, enucleation group. Statistics for **E** (left) and **H** (left) are in the main text. Statistics for (**E**) (right), (**F**), (**G**) and (**H**) (right) are in Table 4.

While AMPAR-containing synapses represent the mature state of glutamatergic synapses, developing cortical circuits also have NMDAR-only synapses, which is a feature of the developing nervous system^44–46^. The fraction of NMDAR-only input to cortical GABAergic interneurons is higher in the first postnatal week than at later ages^23^. Since NMDAR-only inputs can be gradually converted into AMPAR-containing inputs by neural activity during postnatal development^24,45,47^, we tested if enucleation altered the developmental reduction of the NMDAR-only inputs and if such changes mirror the observed changes in the AMPAR-mediated inputs. For each neuron, we performed recordings at holding potentials of − 70 mV and 40 mV during LSPS glutamate uncaging (Fig. 4A). We identified the spatial locations of NMDAR-only inputs as stimulation sites where monosynaptic EPSCs were observed only at 40 mV holding potential but not at − 70 mV (Fig. 4B). For each recorded *Gad2* interneuron we generated an NMDAR-only input location map and then aligned the maps of all cells in each condition at the soma and averaged them, resulting in an input location probability map for NMDAR-only inputs (Fig. 4C). Qualitative inspection showed that few consistent NMDAR-only inputs were present but that the spatial pattern of NMDAR-only inputs to *Gad2* interneurons was similar between control and enucleated animals. The laminar plots revealed a high variance of input pattern from the columnar profile of each cell (Fig. 4D). Quantifying the total number of NMDAR-only inputs showed a similar amount of input between enucleated and control animals (Fig. 4E, left; control median: 6, IQR: 8, enucleation median: 5, IQR: 13, P = 0.817, effect size: r = 0.05, Wilcoxon rank-sum test).

Quantification of the laminar sources of inputs revealed no obvious differences between cells from enucleated and control animals (Fig. 4E, right; Table 4), but small increases were present in the average proportion of inputs from L4 (Fig. 4F; Table 4). Analysis of the correlations of the input locations of NMDAR-only inputs revealed that the location of NMDAR-only inputs from L4 was less variable in the enucleated animals (Fig. 4G; Table 4).

**Table 4:** Statistics in Fig. 4.

Figure 4E: average number of effective stimulus locations in each layer group							
Layer groups	Control		Enucleation		P	Effect size	Test method
	Median	IQR	Median	IQR			
L2/3	1.81	2.12	1.47	7.51	0.756	0.06	Wilcoxon rank-sum test
L4	0.84	2	2.61	3.59	0.109	0.31	Wilcoxon rank-sum test
L5/6	1.8	4.57	1	3.57	0.511	0.13	Wilcoxon rank-sum test

Figure 4F: percentage of input in each layer group							
Layer groups	Control		Enucleation		P	Effect size	Test method
	Median	IQR	Median	IQR			
L2/3	0.396	0.353	0.311	0.127	0.478	0.3	Two-sample *t*-test
L4	0.172	0.347	0.375	0.476	0.031	1.08	Two-sample *t*-test
L5/6	0.536	0.478	0.2	0.419	0.136	0.32	Wilcoxon rank-sum test

Figure 4G: correlation of effective stimulus locations in each layer group							
Layer groups	Control		Enucleation		P	Effect size	Test method
	Median	IQR	Median	IQR			
L2/3	− 0.189	0.427	− 0.063	0.666	0.115	0.17	Wilcoxon rank-sum test
L4	− 0.125	0.384	0.133	0.393	0.032	0.25	Wilcoxon rank-sum test
L5/6	− 0.052	0.619	0.283	0.684	0.127	0.49	Two-sample *t*-test

Figure 4H: NMDAR-only to AMPAR-mediated input ratio							
Layer groups	Control		Enucleation		P	Effect size	Test method
	Median	IQR	Median	IQR			
L2/3	0.133	0.125	0.201	0.844	0.324	0.2	Wilcoxon rank-sum test
L4	0.06	0.269	0.31	0.64	0.109	0.31	Wilcoxon rank-sum test
L5/6	0.146	0.295	0.326	0.503	0.618	0.1	Wilcoxon rank-sum test

*IQR* interquartile range.

We next computed the overall percentage of NMDAR-only input (NMDAR-only input to AMPAR-mediated input ratio) and found that it was comparable between control and enucleation group (Fig. 4H, left; control median: 0.16, IQR: 0.16, enucleation median: 0.2, IQR: 0.53, P = 0.395, effect size: r = 0.17, Wilcoxon rank-sum test). Separately computing the percentage of NMDAR-only input in different layer groups showed that the laminar distribution of NMDAR-only input was comparable between control and enucleation group (Fig. 4H, right; Table 4).

Together our findings showed that the absence of retinal input did not alter NMDAR-only inputs to L4 *Gad2* interneurons in V1 identified by our method. Since we find that enucleation prevents the development of AMPAR-mediated connections, these results suggest that the increase in AMPAR-mediated connections and decrease in NMDAR-only connections might be independently regulated.

### The strength of AMPAR-mediated and NMDAR-only connections in *Gad2* interneurons is not altered after enucleation

**Figure 5:**
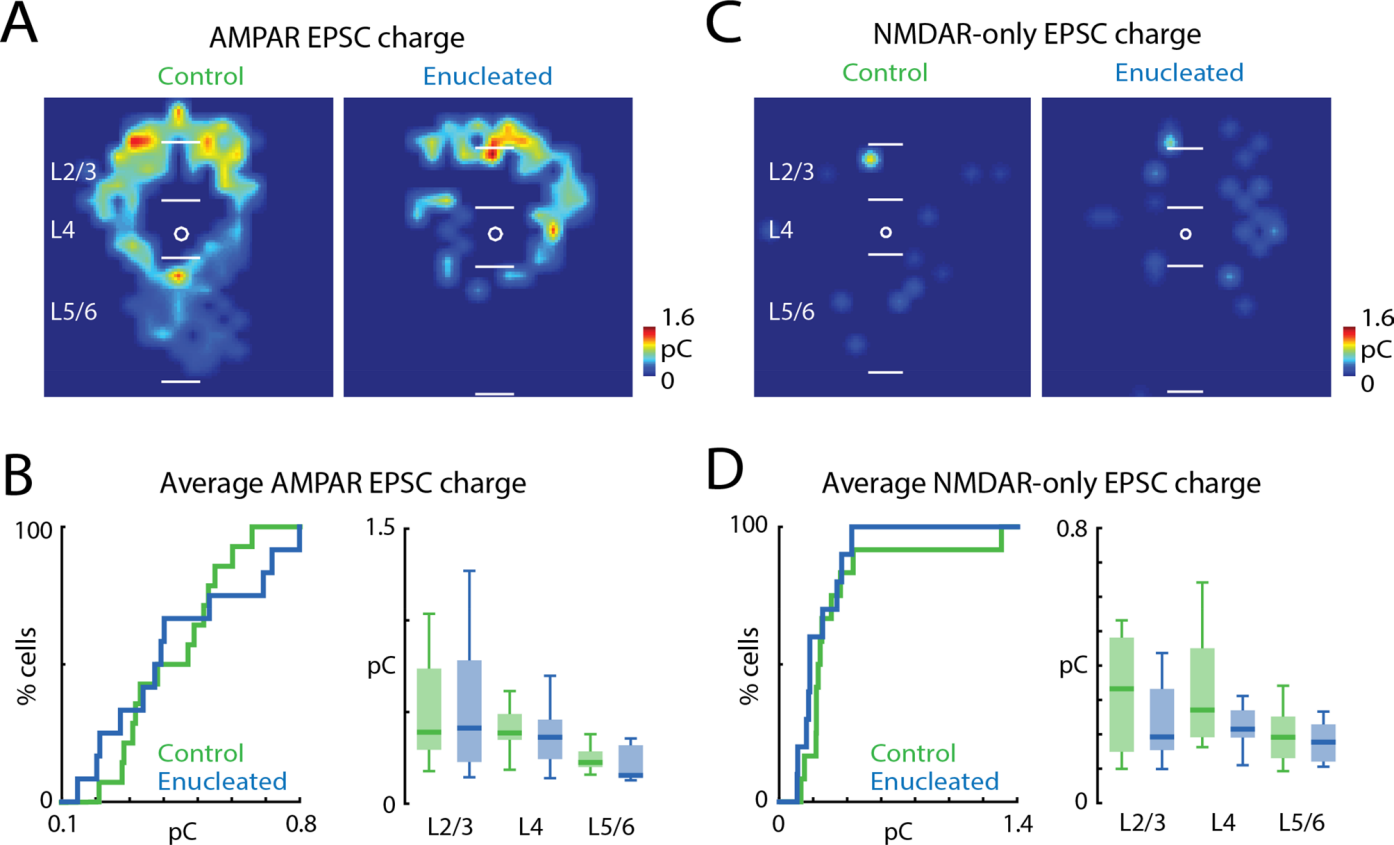
Strength of synaptic connections in *Gad2* interneurons is not altered by enucleation. (**A**) Average EPSC charge of AMPAR-mediated inputs received by *Gad2* interneurons in control and enucleated mice. (**B**) Quantification of AMPAR-mediated EPSC transferred charge. CDF of average transferred charge for each cell (left) and for inputs from each layer group (right). P > 0.05. (**C**) Average EPSC charge of NMDAR-only inputs received by *Gad2* interneurons in control and enucleated mice. (**D**) CDF of average NMDAR-only transferred charge for each cell (left) and for inputs from each layer group (right). P > 0.05. For (**A**) and (**C**), white bars mark layer boundaries. The length of the white bars is 100 µm. Input locations are color-coded based on the average EPSC charge (pseudocolor scale to the lower right of each panel). For all panels: green, control group; blue, enucleation group. Statistics for (**B**) (left) and (**D**) (left) are in the main text. Statistics for (**B**) (right) and (**D**) (right) are in Table 5.

So far, we had investigated the spatial patterns of connections without analyzing the strength of these connections. To investigate if enucleation changes the strength of AMPAR-mediated connections to *Gad2* interneurons, we quantified the average transferred charge of the evoked EPSCs. Computing an average spatial map of the transferred charge of AMPAR-mediated EPSCs (Fig. 5A) revealed a map pattern that was similar to the connection probability maps. The overall average EPSC transferred charge (Fig. 5B, left; control median: 0.43 pC, IQR: 0.22 pC, enucleation median: 0.38 pC, IQR: 0.37 pC, P = 0.999, effect size: Cohen’s d = 0.001, two-sample *t*-test) was similar between conditions. The average EPSC transferred charge in separate layers was also similar between control and enucleation animals (Fig. 5B, right; Table 5).

**Table 5:** Statistics in Fig. 5.

Figure 5B: average EPSC transferred charge in each layer group							
Layer groups	Control		Enucleation		P	Effect size	Test method
	Median (pC)	IQR (pC)	Median (pC)	IQR (pC)			
L2/3	0.39	0.44	0.41	0.55	0.497	0.14	Wilcoxon rank-sum test
L4	0.39	0.14	0.36	0.22	0.396	0.17	Wilcoxon rank-sum test
L5/6	0.23	0.09	0.15	0.17	0.076	0.35	Wilcoxon rank-sum test

Figure 5D: average EPSC transferred charge in each layer group							
Layer groups	Control		Enucleation		P	Effect size	Test method
	Median (pC)	IQR (pC)	Median (pC)	IQR (pC)			
L2/3	0.33	0.33	0.19	0.18	0.494	0.15	Wilcoxon rank-sum test
L4	0.27	0.26	0.22	0.08	0.249	0.61	Two-sample *t*-test
L5/6	0.19	0.12	0.18	0.11	0.544	0.29	Two-sample *t*-test

*IQR* interquartile range.

The average spatial map of the transferred charge of EPSCs from NMDAR-only connections (Fig. 5C) was also similar between conditions. Enucleation did not change the overall transferred charge of NMDAR-only connections (Fig. 5D, left; control median: 0.24 pC, IQR: 0.12 pC, enucleation median: 0.18 pC, IQR: 0.18 pC, P = 0.339, effect size: r = 0.2, Wilcoxon rank-sum test) nor the average EPSC transferred charge from different layers (Fig. 5D, right; Table 5). Together, our results suggest that the synaptic strength of glutamatergic connections received by V1 *Gad2* interneurons is not affected by neonatal enucleation.

### Proportion of AMPAR-mediated inputs from subgranular layers decreased in PV interneurons after enucleation

**Figure 6:**
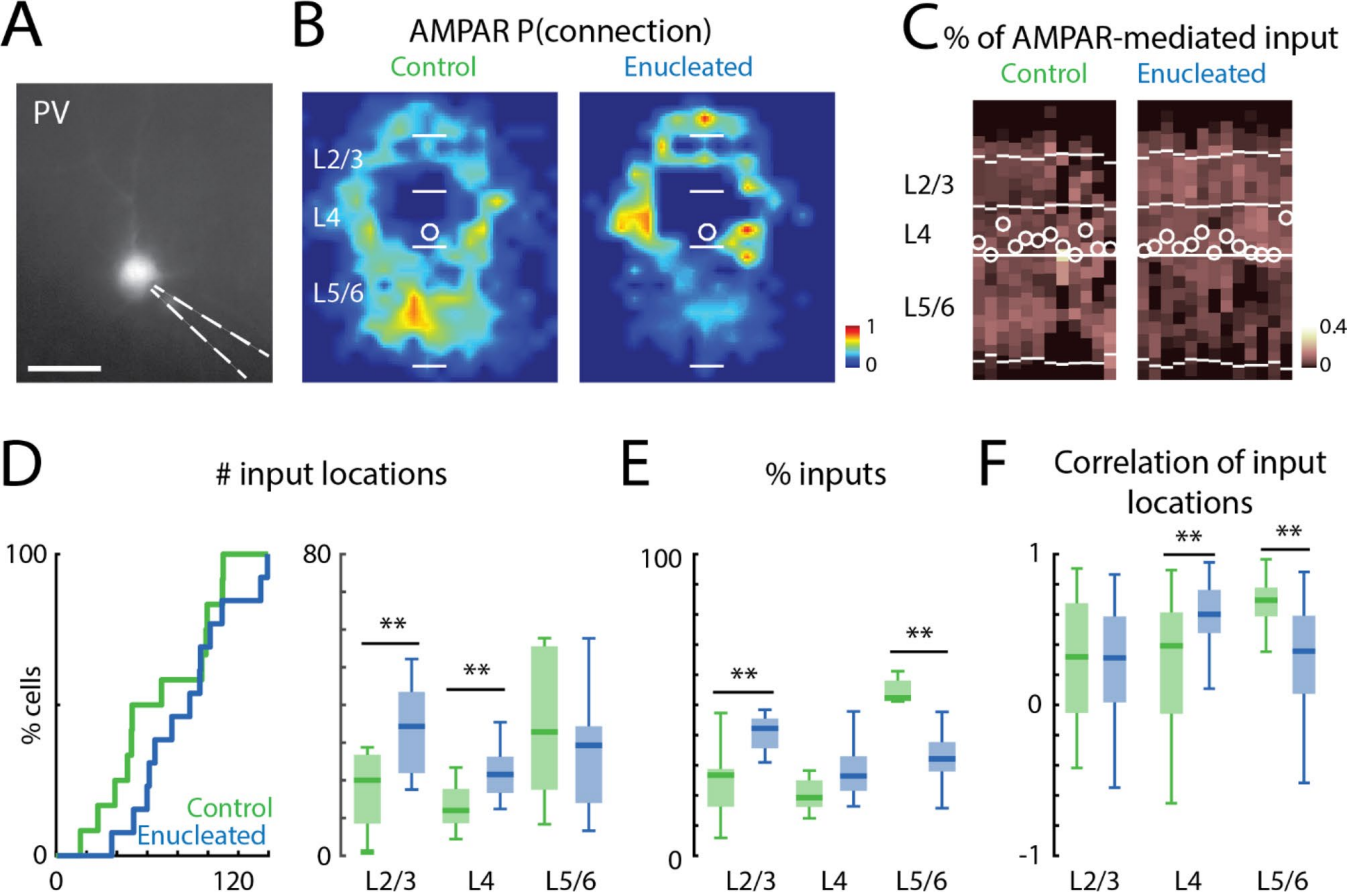
AMPAR-mediated cortical inputs to PV interneurons from supragranular layers are increased after enucleation. (**A**) An example *PV-Cre::tdTomato* interneuron under epifluorescence microscopy. Scale bar = 50 µm. (**B**) Average spatial connection probability maps of AMPAR-mediated inputs to L4 PV interneurons in V1 at P16/P17 in control and enucleated animals (enucleated at P1) (n = 12 control and n = 13 enucleated). Maps show the fraction of cells that received an input from a particular spatial location. The length of the scale bars marking the layer boundaries is 100 μm. (**C**) Laminar distribution of inputs for each cell. Plotted is the fraction of input each cell received at each laminar location. Layer borders for each cell are indicated by horizontal white lines. Soma locations are indicated by white circle; cells are aligned to L4. (**D**) Quantification of the number of input locations. CDF of the total number of input locations (left). Boxplot shows the number of input locations in each layer group (right). AMPAR-mediated inputs from L2/3 and L4 are increased after enucleation. (**) indicates P < 0.01, otherwise P > 0.05. (**E**) Boxplot of the average fraction of inputs from each layer. The fraction of L5/6 inputs is decreased while the fraction of L2/3 inputs is increased after enucleation. (**) indicates P < 0.01, otherwise P > 0.05. (**F**) Boxplot of the correlation of input locations from each layer originate. (**) indicates P < 0.01, otherwise P > 0.05. Statistics for (**D**) (left) are in the main text. Statistics for (**D**) (right), (**E**) and (**F**) are in Table 6.

GABAergic interneurons labeled by *Gad2-Cre* comprise different cell types^30,43^, which may have different developmental trajectory^48–50^ and may respond differently to removal of sensory activity^51^. The results that we observed from *Gad2* interneurons could represent the average of differential changes in different types of GABAergic interneurons. Accordingly, we used a more restricted mouse line—PV-Cre transgenic mouse, to selectively probe changes in fast-spiking, parvalbumin-positive, interneurons^52^ in L4 of V1. PV interneurons play a key role in gating the critical period^25^, thus factors affecting their maturation can influence the later critical period. We thus repeated the above studies in this mouse line and compared AMPAR-mediated and NMDAR-only connections onto PV interneurons (Fig. 6A) at P16/P17 in control and neonatally enucleated mice (control: n = 12 cells, enucleation: n = 13 cells).

Qualitative inspection of the average connection probability maps (Fig. 6B) and laminar input plots (Fig. 6C) showed an increase of AMPAR-mediated inputs from L2–4 and a decrease of AMPAR-mediated inputs from L5/6. Therefore, we quantified the total number of AMPAR-mediate input locations in each PV interneuron. The total number of input locations across all layers was similar between control and enucleation condition (Fig. 6D, left; control median: 59, IQR: 56, enucleation median: 88, IQR: 42, P = 0.181, effect size: Cohen’s d = 0.55, two-sample *t*-test). Since *Gad2* positive interneurons showed laminar specific differences in AMPAR-mediated inputs after enucleation, we performed a laminar analysis. Separately comparing the number of AMPAR-mediated inputs from each layer revealed that PV interneurons from enucleated animals had an increase of AMPAR-mediated inputs from L2/3 and L4 than PV interneurons from control animals (Fig. 6D, right; Table 6). Accordingly, the average proportion of AMAPAR-mediated inputs from L2-4 to PV interneurons was larger in enucleated animals than in control and the fraction of inputs from L5/6 was reduced in enucleated animals (Fig. 6E; Table 6).

**Table 6:** Statistics in Fig. 6.

Figure 6D: average number of effective stimulus locations in each layer group							
Layer groups	Control		Enucleation		P	Effect size	Test method
	Median	IQR	Median	IQR			
L2/3	20	18.2	34.3	21.5	0.0013	1.46	Two-sample *t*-test
L4	12	9	21.5	9.6	0.0032	1.31	Two-sample *t*-test
L5/6	32.8	38	29.3	20.3	0.4014	0.34	Two-sample *t*-test

Figure 6E: percentage of input in each layer group							
Layer groups	Control		Enucleation		P	Effect size	Test method
	Median	IQR	Median	IQR			
L2/3	0.267	0.125	0.423	0.098	4.95 × 10^–4^	1.78	Two-sample *t*-test
L4	0.193	0.088	0.265	0.114	0.107	0.32	Wilcoxon rank-sum test
L5/6	0.524	0.068	0.321	0.097	2.65 × 10^–5^	2.15	Two-sample *t*-test

Figure 6F: correlation of effective stimulus locations in each layer group							
Layer groups	Control		Enucleation		P	Effect size	Test method
	Median	IQR	Median	IQR			
L2/3	0.318	0.728	0.312	0.569	0.602	0.05	Wilcoxon rank-sum test
L4	0.392	0.67	0.601	0.285	1.06 × 10^–5^	0.37	Wilcoxon rank-sum test
L5/6	0.694	0.19	0.357	0.515	6.07 × 10^–13^	0.6	Wilcoxon rank-sum test

*IQR* interquartile range.

Besides increased connectivity from L2-4 in PV cells from enucleated mice, the average connection probability maps had also suggested a difference in the input locations from L5/6. Since the total numbers of connections from L5/6 were similar between cells from enucleated and control mice, such differences in the average maps could be caused by changes in the spatial origins of the inputs. Similar to our approach above, we calculated the correlation of input locations for each layer group. We found that the correlation of input locations was lower for inputs from L5/6 in PV interneurons from enucleated mice (Fig. 6F; Table 6). This larger heterogeneity of L5/6 inputs likely resulted in the observed lower average connection probability from L5/6 in enucleated mice. In contrast to the lower correlation for L5/6 connections we found larger spatial correlation of input originating in L4, suggesting that the locations of the inputs from L4 were more consistent across PV cells from enucleated mice than in control animals.

Together, our results show that after bilateral enucleation the AMPAR-mediated connections from L2/3 to L4 in PV interneurons are increased at P16/P17 and that the spatial pattern of connections from L4 and L5/6 was altered. Thus, similar to our results in *Gad2* interneurons, the maturation and spatial refinement of AMPAR-mediated connections to PV interneurons in L4 of V1 requires retinal input, suggesting that peripheral input might be required for the maturation of different types of GABAergic interneurons.

### NMDAR-only inputs are not altered in PV interneurons after enucleation

**Figure 7:**
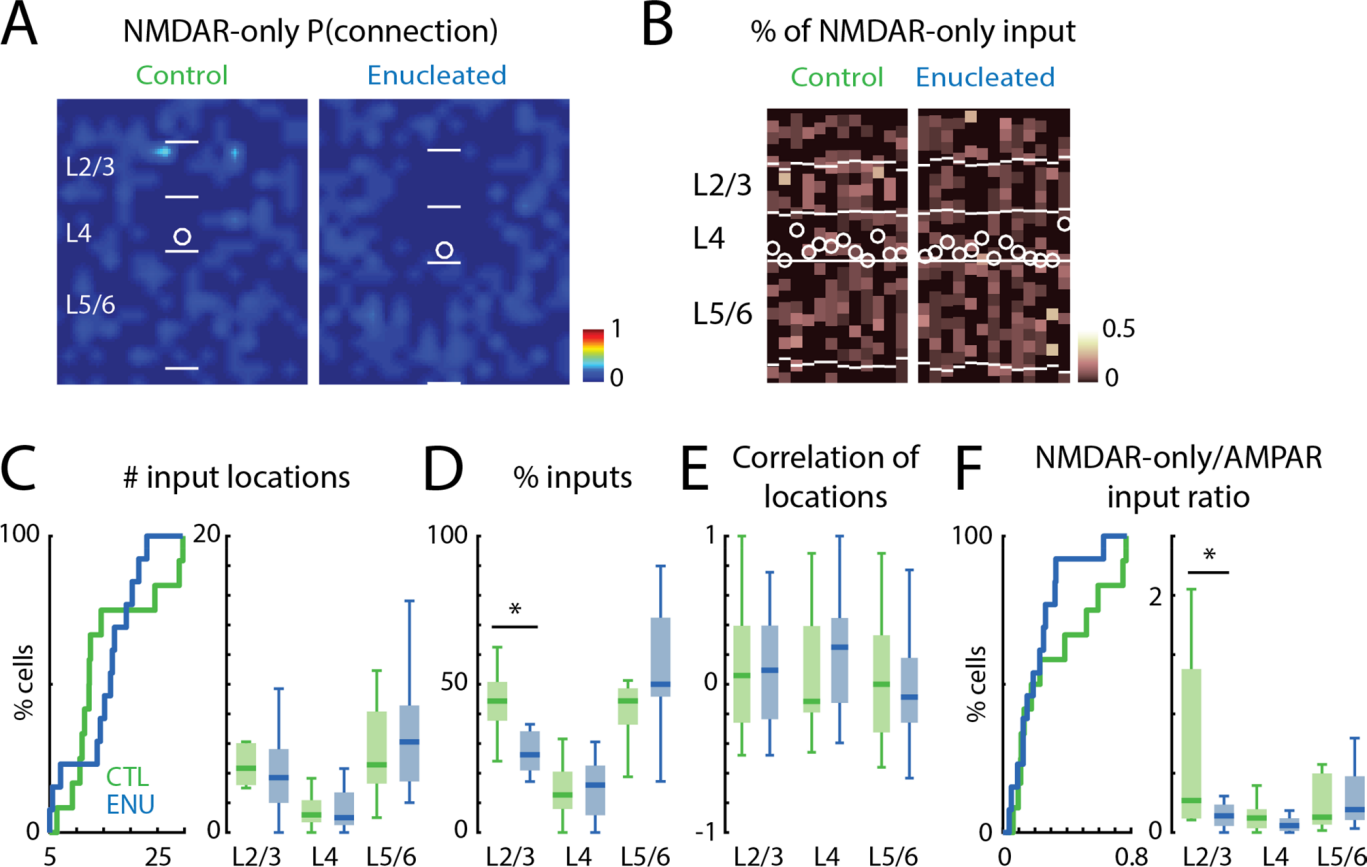
NMDAR-only cortical inputs to PV interneurons. (**A**) Average spatial connection probability maps of NMDAR-only inputs received by L4 PV interneurons in V1 at P16/P17 in control and enucleated animals (n = 12 control and n = 13 enucleated, same cells as in Fig. 6B). (**B**) Laminar distribution of input for each cell. Plotted is the fraction of input each cell received at each laminar location. Layer borders for each cell are indicated by horizontal white lines. Soma locations are indicated by white circle; cells are aligned to L4. (**C**) Quantification of the number of input locations. CDF of the number of input locations (left). Boxplot shows the number of input locations in each layer group (right). P > 0.05. (**D**) Boxplot of the average fraction of inputs from each layer. (*) indicates P < 0.05, otherwise P > 0.05. (**E**) Boxplot of the correlation of input locations from each layer originate. P > 0.05. (**F**) Quantification of the ratio of NMDAR-only inputs to AMPAR-mediated inputs for each cell (left) and for each layer group (right). (*) indicated P < 0.05, otherwise P > 0.05. For all panels: green, control group; blue, enucleation group. Statistics for (**C**) (left) and (**F**) (left) are in the main text. Statistics for (**C**) (right), (**D**), (**E**) and (**F**) (right) are in Table 7.

Binocular enucleation did not alter the NMDAR-only inputs to L4 *Gad2* interneurons identified by our method. Thus, we investigated if NMDAR-only inputs received by PV interneurons were altered after enucleation. We then generated spatial connection probability maps (Fig. 7A) and laminar input plots (Fig. 7B) for NMDAR-only inputs to PV interneurons. Qualitative inspection showed that few NMDAR-only inputs were present but that the laminar pattern of NMDAR-only inputs was highly variable. Quantifying the total number of NMDAR-only inputs showed no significant changes in the number of input locations in PV cells from control and enucleated animals (Fig. 7C, left; control median: 12, IQR: 8, enucleation median: 16, IQR: 7, P = 0.568, effect size: r = 0.11, Wilcoxon rank-sum test). Quantifying of the laminar sources of inputs showed that PV cells from enucleated and control animals received inputs from a similar number of locations from each layer (Fig. 7C, right; Table 7), but that the average proportion of NMDAR-only inputs from L2/3 was decreased (Fig. 7D; Table 7). Analyzing the correlations of the input locations of NMDAR-only inputs revealed that the location of NMDAR-only inputs had similar variance in the control and enucleated animals (Fig. 7E; Table 7).

**Table 7:** Statistics in Fig. 7.

Figure 7C: average number of effective stimulus locations in each layer group							
Layer groups	Control		Enucleation		P	Effect size	Test method
	Median	IQR	Median	IQR			
L2/3	4.33	2.82	3.7	3.63	0.242	0.23	Wilcoxon rank-sum test
L4	1.19	1.47	1	2.2	0.978	0.006	Wilcoxon rank-sum test
L5/6	4.57	4.86	6.12	5.12	0.399	0.34	Two-sample *t*-test

Figure 7D: percentage of input in each layer group							
Layer groups	Control		Enucleation		P	Effect size	Test method
	median	IQR	median	IQR			
L2/3	0.444	0.13	0.263	0.132	0.038	0.87	Two-sample *t*-test
L4	0.127	0.126	0.16	0.167	0.802	0.1	Two-sample *t*-test
L5/6	0.444	0.122	0.5	0.266	0.057	0.79	Two-sample *t*-test

Figure 7E: correlation of effective stimulus locations in each layer group							
Layer groups	Control		Enucleation		P	Effect size	Test method
	Median	IQR	Median	IQR			
L2/3	0.059	0.653	0.094	0.63	0.539	0.06	Wilcoxon rank-sum test
L4	− 0.114	0.582	0.25	0.572	0.055	0.17	Wilcoxon rank-sum test
L5/6	0	0.654	− 0.086	0.436	0.924	0.008	Wilcoxon rank-sum test

Figure 7F: NMDAR-only to AMPAR-mediated input ratio							
Layer groups	Control		Enucleation		P	Effect size	Test method
	Median	IQR	Median	IQR			
L2/3	0.27	1.26	0.14	0.18	0.024	0.45	Wilcoxon rank-sum test
L4	0.123	0.16	0.059	0.105	0.242	0.23	Wilcoxon rank-sum test
L5/6	0.13	0.431	0.194	0.368	0.568	0.11	Wilcoxon rank-sum test

*IQR* interquartile range.

Calculating the overall fraction of NMDAR-only input revealed that the overall NMDAR-only input to AMPAR input ratio is comparable between control and enucleation group (Fig. 7F, left; control median: 0.2, IQR: 0.44, enucleation median: 0.18, IQR: 0.16, P = 0.462, effect size: r = 0.15, Wilcoxon rank-sum test), but that the NMDAR-only input to AMPAR input ratio was slightly decreased in L2/3 (Fig. 7F, right; Table 7).

Together, our data suggest that retinae are not required for the overall development of NMDAR-only inputs to L4 PV interneurons in V1 by P16/P17, but that enucleation might have a slight effect on inputs from L2/3.

### The strength of AMPAR-mediated and NMDAR-only connections in PV interneurons is not altered after enucleation

**Figure 8:**
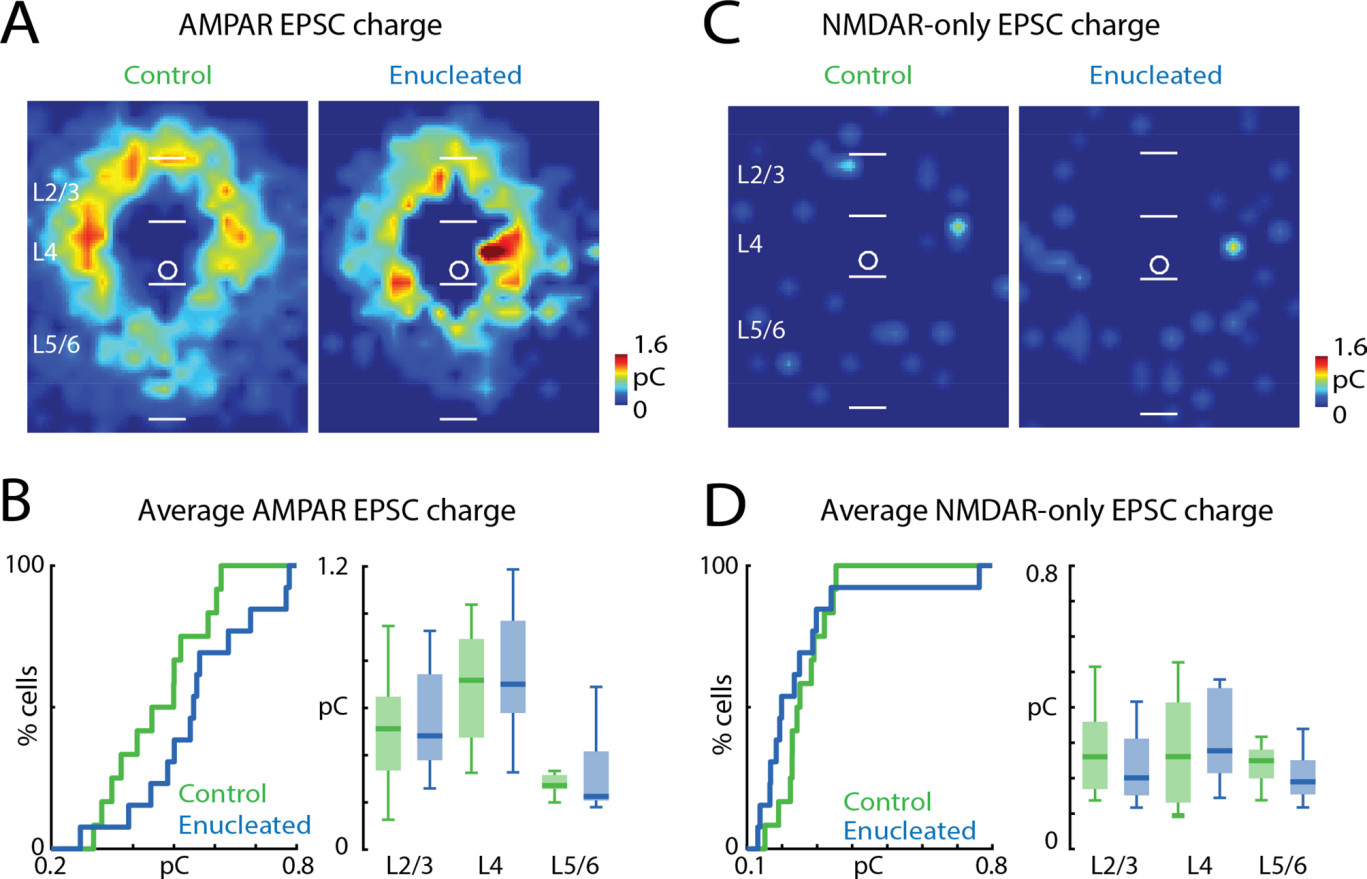
Strength of synaptic connections in PV interneurons is not altered by enucleation. (**A**) Average EPSC charge of AMPAR-mediated inputs received by PV interneurons in control and enucleated mice. (**B**) Quantification of AMPAR-mediated EPSC transferred charge. CDF of average transferred charge for each cell (left) and for inputs from each layer group (right). P > 0.05. (**C**) Average EPSC charge of NMDAR-only inputs received by PV interneurons in control and enucleated mice. (**D**) Quantification of NMDAR-only EPSC transferred charge. CDF of average transferred charge for each cell (left) and for inputs from each layer group (right). P > 0.05. For (**A**) and (**C**), white bars mark layer boundaries. The length of the white bars is 100 µm. Input locations are color-coded based on the average EPSC charge (pseudocolor scale to the lower right of each panel). For all panels: green, control group; blue, enucleation group. Statistics for (**B**) (left) and (**D**) (left) are in the main text. Statistics for (**B**) (right) and (**D**) (right) are in Table 8.

We next investigated if the strength of AMPAR-mediated inputs received by PV interneurons was affected by enucleation. Computing an average spatial map of AMPAR-mediated EPSC transferred charge revealed a pattern that was similar to the connection probability maps (Fig. 8A). The average EPSC transferred charge (Fig. 8B, left; control median: 0.47 pC, IQR: 0.19 pC, enucleation median: 0.55 pC, IQR: 0.17 pC, P = 0.086, effect size: Cohen’s d = 0.71, two-sample *t*-test) of the evoked EPSCs was not changed. The average EPSC transferred charge in each layer was also similar between control and enucleated animals (Fig. 8B, right; Table 8). The average spatial map of the transferred charge of NMDAR-only EPSCs also showed no obvious differences between conditions (Fig. 8C). The average EPSC transferred charge (Fig. 8D, left; control median: 0.25 pC, IQR: 0.08 pC, enucleation median: 0.2 pC, IQR: 0.26 pC, P = 0.265, effect size: r = 0.22, Wilcoxon rank-sum test) was similar between PV cells from enucleated and control animals. When separated into different layers, the average EPSC transferred charge was also similar between the control and enucleated animals (Fig. 8D, right; Table 8). Thus, early removal of retinae has no effect on the strength of either AMPAR-mediated or NMDAR-only inputs to PV interneurons.

**Table 8:** Statistics in Fig. 8.

Figure 8B: average EPSC transferred charge in each layer group							
Layer groups	Control		Enucleation		P	Effect size	Test method
	Median (pC)	IQR (pC)	Median (pC)	IQR (pC)			
L2/3	0.51	0.31	0.48	0.36	0.714	0.15	Two-sample *t*-test
L4	0.72	0.42	0.7	0.38	0.57	0.23	Two-sample *t*-test
L5/6	0.27	0.06	0.23	0.21	0.497	0.14	Wilcoxon rank-sum test

Figure 8D: average EPSC transferred charge in each layer group							
Layer groups	Control		Enucleation		P	Effect size	Test method
	Median (pC)	IQR (pC)	Median (pC)	IQR (pC)			
L2/3	0.26	0.19	0.2	0.16	0.258	0.48	two-sample *t*-test
L4	0.26	0.28	0.28	0.24	0.47	0.15	Wilcoxon rank-sum test
L5/6	0.25	0.08	0.19	0.1	0.15	0.29	Wilcoxon rank-sum test

*IQR* interquartile range.

## Discussion

Our results reveal that retinae are required for the normal development of AMPAR-mediated glutamatergic connections received by L4 GABAergic interneurons in the V1 during the pre-critical period of development. We show that in both *Gad2*- and PV-expressing interneurons the proportion of AMPAR-mediated inputs from L5/6 was reduced after enucleation, suggesting a similar dependence of glutamatergic circuit maturation on retinal input across different types of GABAergic interneurons. Thus, as a result of early retinal removal the translaminar organization of AMPAR-mediated circuit to L4 GABAergic neurons is shifted towards receiving more inputs from L2/3. However, *Gad2* interneurons showed fewer inputs from L5/6 after enucleation while PV interneurons showed an overabundance of connections from L2/3. Moreover, these changes in the number of connections were accompanied by changes in the spatial pattern of connections. Thus, peripheral retinal input is required for the maturation of the translaminar organization of AMPAR-mediated circuits to L4 GABAergic interneurons and the spatial patterning of these circuits. In contrast to the changes in AMPAR-mediated connections, NMDAR-only connections identified by our method were largely unaffected by enucleation.

Our finding that the development of AMPAR-mediated connections to GABAergic *Gad2* and PV interneurons in V1 is affected by peripheral retinal input is consistent with prior studies of excitatory inputs to GABAergic interneurons^23,51^. In rodents during the first two postnatal weeks, connections from cortical glutamatergic to GABAergic interneurons transition from initially being dominated by NMDAR-only synapses towards AMPAR-containing synapses^21–23^. Removal of cochlea changed the spatial distribution of AMPAR-mediated connections from L2/3 and L5/6 to *Gad2* interneurons in the auditory cortex. Connections from L2/3 became more widespread while connections from L5/6 became more spatially restricted^23^. Blocking NMDAR-mediated thalamic activity in Reelin interneurons resulted in a loss of thalamic but increase in intracortical connectivity^51^, suggesting a peripheral activity-dependent reorganization of inputs. Our results here, showing that the loss of peripheral retinal input results in a laminar reorganization of AMPAR-mediated connections in V1, are consistent with these results. Thus, peripheral input is important for the normal development of the cortical GABAergic interneurons. However, due to the direct activation of the target cell by photolysis, our method cannot resolve connections within about 100 μm of the recorded neurons. It thus remains unknown if there are any changes to these very local connections after binocular enucleation.

Glutamatergic synapses often transition from a NMDAR-only state to an AMPAR-containing state during maturation^44,46,47^. This transition is associated with the closure of ocular dominance plasticity in the visual cortex^53,54^. We find that NMDAR-only connections are largely unaffected by peripheral manipulations, suggesting that NMDAR-only connections are regulated independently of AMPAR-mediated connections. However, our method can only detect NMDAR-only connections from locations distinct from the locations with AMPAR-mediated connections, so the NMDAR-only inputs we detected were underestimated. We cannot rule out the possible changes of NMDAR-only inputs that are spatially located within the range of AMPAR-containing inputs—e.g., if presynaptic neurons in a particular location signal to the GABAergic interneuron under study via both AMPAR-containing and NMDAR-only synapses. Nevertheless, our results do suggest that the impact of enucleation is mainly on AMPAR-mediated connections.

Our results also showed that the excitatory neural networks innervating *Gad2* and PV interneurons was differentially impacted by enucleation. We found that after enucleation L4 *Gad2* interneurons received fewer AMPAR-mediated inputs from L5/6, while L4 PV interneurons received more AMPAR-mediated inputs from L2/3 and L4. The analysis of the spatial correlation of input locations revealed additional changes of the presynaptic locations in the presynaptic layers that did not show overt changes in input number in both *Gad2* and PV interneurons. The locations of AMPAR-mediated inputs received by *Gad2* interneurons became more heterogeneous, while for PV interneurons, the heterogeneity of the locations of AMPAR-mediated inputs decreased in L4 and increased in L5/6 after enucleation. The differences between *Gad2* and PV interneurons likely reflect the heterogeneity of the *Gad2* positive population^32,43^. GABAergic interneurons have diverse morphologies, electrical properties and synaptic connectivity^55,56^. Different GABAergic interneurons can vary in their developmental trajectory^48–50^, and respond differently to the same manipulation^51,57^. In contrast to the *Gad2*-positive population, the PV-Cre-labeled interneurons specifically encompass fast spiking interneurons^52^. Our results show that for both *Gad2* and PV interneurons, binocular enucleation resulted in an increased proportion of AMPAR-mediated inputs from L2/3 and decreased proportion of AMPAR-mediated inputs from L5/6. These observations indicate that the development of cortical subgranular circuits and subgranular circuits might be differentially impacted by early removal of retinal input and thus might be differentially regulated. Subgranular layers encompass L6, which can be thalamorecipient^58–65^ and also contain upper subplate neurons^66–68^. This suggests that pathways relaying peripheral activity engage these subgranular circuits and that subgranular circuits might play a key role during development.

Enucleation, widely used to study the influence of the sensory periphery on central visual circuits^69–72^, removes both early spontaneous and later visually driven retinal activity. In our experiments, binocular enucleation was done at P1/P2 and cortical glutamatergic inputs received by GABAergic interneurons were tested at P16/P17, when V1 activity patterns have matured^29^. This time period is before the onset of the ocular dominance plasticity in V1^40–42^ but after eye opening. Before eye opening, the retina produces spontaneous activity^73–76^. At later stages and especially after eye opening retinal activity can be light-driven^29,77^. During this time period, retinal activity can affect neural activity in the developing visual cortex^29,78–81^ and thus can contribute to the development of the visual cortex^5,82,83^. Thus, both spontaneous retinal waves and light-driven retinal activity could contribute to the development of AMPAR-mediated inputs received by L4 *Gad2* and PV interneurons in V1. Further studies using selective manipulations of either spontaneous or light-evoked retinal activity are needed to evaluate the contributions of different sources of activity.

It is still unknown how much of the activity in V1 is changed at P16/17 after neonatal enucleation. In adult mice, after losing retinal input, neural activity in V1 rapidly decreases, recovering within 72 h due to homeostatic plasticity^70,84^. However, the activity in cortical inhibitory neurons does not recover within 72 h^85^, suggesting a compensatory decrease of cortical inhibition to decreased activity from retina. Similar homeostatic changes could happen in the neonatal enucleation model during early postnatal development. Peripheral manipulations can cause changes at multiple levels of processing, making direct inferences about the mechanisms underlying the effects we observe difficult. For example, correlations in LGN activity increase after optic nerve cuts^86^ and such changes could at least partially compensate for retinal loss. Thus, changes seen on the cortical level could be due to changed average activity, changed correlations, or both in thalamocortical and possibly corticothalamic circuits. Nevertheless, our results show that the retina is required for the maturation of AMPAR-mediated connections onto GABergic interneurons.

Peripheral input does not only influence GABAeric interneurons at early ages. Later manipulations of sensory activity can further change these connections. For example, monocular deprivation at P28/29 reduces the AMPAR-mediated inputs to PV interneurons in V1^87^. In particular, these studies showed that reduced visual activity led to decreased numbers of inputs and decreased amplitude of excitatory L4 and L5a input to L2/3 PV interneurons. Together with our data these results suggest that peripheral retinal input has a powerful influence on the laminar distribution of excitatory inputs onto inhibitory neurons over development, starting at the earliest postnatal ages.

In summary, our results show that early retinal input can shape the functional connectivity impinging on GABAergic interneurons. It is likely that early effects can alter the subsequent trajectory of cortical development.
